# Hydroxyapatite-Coated Titanium by Micro-Arc Oxidation and Steam–Hydrothermal Treatment Promotes Osseointegration

**DOI:** 10.3389/fbioe.2021.625877

**Published:** 2021-08-19

**Authors:** Xiaojun Wang, Lina Mei, Xuesheng Jiang, Mingchao Jin, Yan Xu, Jianyou Li, Xiongfeng Li, Zhipeng Meng, Junkun Zhu, Fengfeng Wu

**Affiliations:** ^1^Department of Orthopedics, Huzhou Central Hospital, Affiliated Central Hospital Huzhou University, Zhejiang University Huzhou Hospital, Huzhou, China; ^2^Department of Orthopedics, Huzhou Traditional Chinese Medicine Hospital, Affiliated to Zhejiang Chinese Medical University, Huzhou, China; ^3^Internal Medicine, Huzhou Maternity and Child Health Care Hospital, Huzhou, China; ^4^Department of Rehabilitation, Huzhou Central Hospital, Affiliated Central Hospital Huzhou University, Zhejiang University Huzhou Hospital, Huzhou, China; ^5^Department of Anaesthesiology, Huzhou Central Hospital, Affiliated Central Hospital Huzhou University, Zhejiang University Huzhou Hospital, Huzhou, China; ^6^Orthopedics Rehabilitation Department, Lishui Municipal Central Hospital, Lishui, China

**Keywords:** micro-arc oxidation, osteogenesis, angiogenesis, osteoimmune microenvironment, osseointegration

## Abstract

Titanium (Ti)-based alloys are widely used in tissue regeneration with advantages of improved biocompatibility, high mechanical strength, corrosion resistance, and cell attachment. To obtain bioactive bone–implant interfaces with enhanced osteogenic capacity, various methods have been developed to modify the surface physicochemical properties of bio-inert Ti and Ti alloys. Nano-structured hydroxyapatite (HA) formed by micro-arc oxidation (MAO) is a synthetic material, which could facilitate osteoconductivity, osteoinductivity, and angiogenesis on the Ti surface. In this paper, we applied MAO and steam–hydrothermal treatment (SHT) to produce HA-coated Ti, hereafter called Ti–M–H. The surface morphology of Ti–M–H1 was observed by scanning electron microscopy (SEM), and the element composition and the roughness of Ti–M–H1 were analyzed by energy-dispersive X-ray analysis, an X-ray diffractometer (XRD), and Bruker stylus profiler, demonstrating the deposition of nano-HA particles on Ti surfaces that were composed of Ca, P, Ti, and O. Then, the role of Ti–M–H in osteogenesis and angiogenesis *in vitro* was evaluated. The data illustrated that Ti–M–H1 showed a good compatibility with osteoblasts (OBs), which promoted adhesion, spreading, and proliferation. Additionally, the secretion of ALP, Col-1, and extracellular matrix mineralization was increased by OBs treated with Ti–M–H1. Ti–M–H1 could stimulate endothelial cells to secrete vascular endothelial growth factor and promote the formation of capillary-like networks. Next, it was revealed that Ti–M–H1 also suppressed inflammation by activating macrophages, while releasing multiple active factors to mediate osteogenesis and angiogenesis. Finally, *in vivo* results uncovered that Ti–M–H1 facilitated a higher bone-to-implant interface and was more attractive for the dendrites, which promoted osseointegration. In summary, MAO and SHT-treated Ti–M–H1 not only promotes *in vitro* osteogenesis and angiogenesis but also induces M2 macrophages to regulate the immune environment, which enhances the crosstalk between osteogenesis and angiogenesis and ultimately accelerates the process of osseointegration *in vivo*.

## Introduction

Regeneration-related tissue engineering has been implicated in many aspects of tissue transplantation to regain the biological function of a tissue or entire organ (Wobma and Vunjak-Novakovic, 2016). Besides this, bone graft ranks as the second most common tissue transplantation (Oryan et al., 2014). Some key factors that are responsible for an ideal and successful bone graft should be taken into account, such as graft cost and feasibility, biological material, biomechanical and morphological characteristics, graft viability, bone integration or osseointegration, ethical issues, etc. (Brydone et al., 2010; Fillingham and Jacobs, 2016; Sohn and Oh, 2019). Allografts and xenografts are easier to obtain, whereas they lack the ability of osteogenesis compared with autografts (Kao et al., 2005). Although autografts are considered as the “gold standard” of bone transplantation, they do have some disadvantages, including potential donor site morbidity and vessel injuries, which constrain their application in clinics (Gulick and Yoder, 2002; Lavender et al., 2020). Thus, in recent decades, researchers have introduced biological or synthetic tissue engineering including, but not limited to, the use of bioactive scaffolds, growth factors, stem cells, and three-dimensional (3D) bioprinting to replace the three grafts mentioned above, which are applied to repair and regenerate bone tissues (Khademhosseini and Langer, 2016).

Titanium (Ti)-based alloys are widely used in tissue regeneration with advantages of improved biocompatibility, high mechanical strength, corrosion resistance, and cell attachment (Jiang et al., 2019). Tβ–Ti alloys can serve as promising new-generation biomedical implant materials for their low Young’s modulus and excellent biocompatibility (Luo et al., 2020). However, a raw Ti transplant without any modification is nearly inert such that it fails to achieve osseointegration *in vivo* (Yu et al., 2014). To obtain bioactive bone–implant interfaces with enhanced osteogenic capacity, various methods have been developed to modify the surface physicochemical properties of bio-inert Ti and Ti alloys (Mendonca et al., 2009; Pierre et al., 2019). Thus, to better apply Ti-based transplant into bone regeneration, Ti surface modification, such as through micro-arc oxidation (MAO), has been introduced to change the physicochemical composition and to improve its biological performance (Sobolev et al., 2018; Wang et al., 2019).

Micro-arc oxidation can generate a porous nano-structure on the surface of Ti, which has been reported to increase the osseointegration capability (Li et al., 2004; Zhou et al., 2017). Nano-structured hydroxyapatite (HA) formed by MAO is a synthetic material which is used as scaffolds and has been shown to facilitate osteoconductivity, osteoinductivity, and angiogenesis on the Ti surface (Patlolla and Arinzeh, 2014; Malhotra and Habibovic, 2016; Shbeh et al., 2019). The nano-HA structure enables the porous scaffold to grow osteoblasts (OBs) and to facilitate angiogenesis by endothelial cells (ECs). However, small pore sizes may inhibit cell growth, production of the extracellular matrix (ECM), and neovascularization on the scaffold (Oryan et al., 2014), which indicates that a successful bone transplantation depends on the controllable nano-HA scaffold. Bai et al. (2018) have elucidated that steam–hydrothermal treatment (SHT) could control the porous sizes of the nano-structure formed on a Ti surface, which does affect osseointegration. Ti nanotube arrays that were fabricated on titanium substrates were adopted for antibacterial drug loading for osteogenesis and anti-bacteria effects (Liu et al., 2017).

Bone repair consists of complicated processes, including the inflammatory response in the microenvironment, which plays pivotal roles in *de novo* bone formation (Oryan et al., 2014, 2017). Therefore, the interaction between the bone graft and the surrounding mediators such as macrophages should be taken into account. In this paper, we not only applied MAO and SHT to modify the morphology of Ti and investigated how this Ti–M nano-structure benefits osseointegration but also discussed the crosstalk among macrophages, OBs, and ECs to further elucidate the mechanism by which Ti–M coating induced *de novo* bone formation.

## Materials and Methods

### Titanium Specimen Preparation

The Ti foil (0.2 mm, 99.6% purity) was trimmed to a square (10 mm × 10 mm) for *in vitro* studies. The pure Ti rod (3 mm in diameter, 99.6% purity) was trimmed to 5 mm in length for *in vivo* experiments. The sample was sonicated and washed in the order of acetone, absolute alcohol, and deionized water (DiH_2_O) for 10 min, followed by air-drying to prepare for surface modifications.

### Surface Modification of Ti Samples

The Ti surface was modified by MAO. The pure Ti served as the anode, while a stainless steel reaction cell was used as the cathode. Next, 0.2 M calcium acetate (C_4_H_6_CaO_4_) and 0.04 M β-glycerophosphate disodium salt pentahydrate (C_3_H_7_Na_2_O_6_P⋅5H_2_O) were dissolved in DiH_2_O to prepare the electrolyte. Then, the current, pulse frequency, duty cycle, and oxidization time were set up as follows: 0.3 A, 800 Hz, 26%, and 5 min, respectively. The modified sample was named Ti–M. Next, the sample was subjected to SHT, by which Ti–M was placed on a pure Ti holder in a Teflon reactor (volume, 40 ml) with the addition of 10 ml ultra-pure water. The Teflon reactor was autoclaved at 250°C for 1, 4, and 8 h and named as Ti–M–H1, Ti–M–H4, and Ti–M–H8, respectively, depending on the time of the SHT.

### Morphology and Characterization of Surface Structure

The sample morphologies of the surface and cross-sections were evaluated through field-emission scanning electron microscopy (FE-SEM) (JSM-7001F, JEOL, Tokyo, Japan). Then, energy-dispersive X-ray analysis (EDX, QX200, Bruker PharmaScan, Ettlingen, Germany) was applied to analyze the distributions of Ca, P, Ti, and O in the cross-sections. Next, the Digimizer software was used to assess the size of surface nano-structures, and the Bruker stylus profiler (Bruker PharmaScan) was conducted to analyze the average surface roughness (Ra) of the sample. Finally, the crystal structure of the sample surface was characterized by an XRD.

### Measurement of Surface Wettability

In order to determine the wettability of the sample surface, 2 μl of DiH_2_O or Dulbecco’s modified Eagle’s medium (DMEM) (Life Technologies Corporation, Gaithersburg, MD, United States) was deposited on the surface of each sample. A contact angle analyzer (Solon, United States) was used to measure the static contact angle *via* the pendant drop method at room temperature.

### Ion Release Measurement

To measure the release of Ca and P ions, inductively coupled plasma-mass spectrometry (Agilent 7500, Agilent Technologies, Santa Clara, CA, United States) was applied to analyze the ions released from the sample immersed in phosphate-buffered saline (PBS).

### Cell Culture

MC3T3-E1 cells (OBs), pre-osteoblasts, were obtained from the cell bank of the Chinese Academy of Sciences (catalog number: GNM15), which were cultured in α-modified minimum essential medium (α-MEM) supplemented with 10% fetal bovine serum (FBS), 100 μg/ml streptomycin, 100 U/ml penicillin, and 0.25 μg/ml amphotericin B in a 37°C incubator with 5% CO_2_. The α-MEM was removed and refreshed every 2 days. After culturing for 3 days, the osteogenic differentiation of OBs was induced by culturing in osteogenic medium (normal medium with the addition of 10 mM β-glycerophosphate, 50 μg/ml ascorbic acid, and 10 nM dexamethasone). OBs were seeded on the surface of the sample at a density of 2 × 10^4^ cells/cm^2^ without specific instruction.

### Adhesion of OBs and Cytoskeletal Assembly on the Sample

The OBs were seeded on the surfaces of different samples and cultured in the incubator of 0.5, 1, and 4 h. Then, the OBs were washed three times with PBS, followed by fixation with 4% paraformaldehyde (PFA) and 4,6-diamidino-2-phenylindole (DAPI, Sigma-Aldrich, St. Louis, MO, United States) staining for 10 min. Confocal laser scanning microscopy (CLSM, C2 Plus, Nikon, Tokyo, Japan) was applied to capture the cell fluorescent images by randomly selecting six fields under × 10 magnification.

The seeded and fixed OBs for 24 h were stained with FITC-phalloidin (Sigma-Aldrich) and counterstained with DAPI, from which the fluorescent images were captured under × 20 magnification by CLSM.

### FE-SEM to Analyze the Morphology of OBs

The OBs were seeded on the surface of the sample for 1 day, followed by washing three times with PBS and fixation with 2.5% glutaraldehyde at 4°C for 1 h. Then, the sample was dehydrated in a gradient of ethanol and air-dried for 1 h, gold-coated, and visualized by FE-SEM to observe the pseudopodia spread of OBs.

### Cytotoxicity Analysis of OBs

A Live/Dead Viability/Cytotoxicity kit (Invitrogen, Carlsbad, CA, United States) was used to qualitatively detect the viability of OBs on the Ti–M–H sample. The OBs were seeded on the surface of the sample for 1, 3, and 5 days. At each time point, the sample was washed three times with PBS, followed by addition of 50 μl working solution (Live/Dead Viability/Cytotoxicity kit, Invitrogen). After incubation in the dark for 1 h, the sample was rinsed gently with PBS, and live cells stained in green and dead cells stained in red were visualized *via* CLSM.

### Proliferation of OBs

A 3-(4, 5-Dimethylthiazol-2-yl)-2,5-diphenyltetrazolium bromide (MTT) assay was performed to analyze the proliferation of OBs on the surface of Ti–M–H. The OBs were seeded on the surface of the sample for 1, 3, and 5 days and washed with PBS. After that, the cells were added with 900 μl normal medium and 10 μl 5 mg/ml MTT and then cultured for 4 h, followed by rinsing with PBS and addition of 1 ml dimethyl sulfoxide. Finally, 100 μl of each sample was transferred to a 96-well plate, which was subjected to a microplate reader (TECAN, Männedorf, Switzerland) to measure the optical density (OD) at 490 nm.

### Alkaline Phosphatase Activity Analysis

The ALP activity of OBs was analyzed by an ALP Color Development Kit (Beyotime, Shanghai, China). First, OBs were seeded on the surface of the sample, and the medium was renewed by osteogenic induction medium after 3 days. After induction for 3 days, the cells were washed with PBS and fixed with 4% PFA for 30 min. The OBs were stained according to the instructions provided by the BCIP/NBT colorimetric kit, and pictures were captured using an inverted microscope (Zeiss, Jena, Germany). The staining area was quantified by Image J 1.45 software.

### Collagen Secretion Analysis of OBs

The level of collagen secretion by OBs on the sample surface was assessed *via* Direct Red 80 (Sigma-Aldrich) staining. The OBs were seeded on the sample surface, and the medium was renewed by osteogenic induction medium after 3 days. After induction for 7 and 14 days, the cells were washed with PBS, fixed with 4% PFA for 30 min, and stained with 0.1 wt% Direct Red 80 for 18 h at room temperature. The sample was washed thoroughly with 0.1 M acetic acid and de-stained with 1 ml 0.2 M NaOH/methanol (1:1). Besides this, 150 μl of staining solution was aliquoted into a 96-well plate for the measurement of OD values at 570 nm.

### Mineralization Assay of Extracellular Matrix

Alizarin Red S (Sigma-Aldrich) was applied to stain the ECM mineralization of OBs. The OBs were washed three times with PBS 7 and 14 days after osteogenic induction, followed by fixation in 75% ethanol for 1 h and staining in 40 mM Alizarin Red S for 30 min at room temperature. The extra staining solution was washed away by distilled water, and 500 μl 10 mM sodium phosphate (pH 7.0) with 10% cetylpyridinium chloride was used to wash away the adsorbed stain for the quantitative analysis. OD values were measured using a microplate reader at 540 nm.

### Endothelial Cell Culture

The ECs used in this study were human umbilical vein fusion cells, EA.hy926, which were purchased from the cell bank of the Chinese Academy of Sciences (catalog number: GNHu39). The EA.hy926 cells were cultured in DMEM supplemented with 10% FBS, 100 U/ml penicillin, 100 μg/ml streptomycin, and 0.25 μg/ml amphotericin B in a 37°C incubator. The medium was refreshed every 2 days.

### EC Vitality and Functionality on the Sample Surface

After the cells were cultured on the sample surface for 1, 3, and 5 days, the viability of ECs was analyzed as described in “Cytotoxicity Analysis of OBs”. After culturing on the sample surface for 3 and 7 days, the enzyme-linked immunosorbent assay (ELISA) (R&D Systems, Minneapolis, MN, United States) was performed to measure the level of vascular endothelial growth factor (VEGF) secreted by ECs according to the manufacturer’s protocols, by which the results were normalized to the number of cells. The capillary tube formation of ECs was assessed by *in vitro* ECMatrix^TM^ gel kit (Millipore, Billerica, MA, United States). An inverted light microscope (Zeiss) was used to capture the images of capillary-like networks from six randomly selected fields. Image J 1.45 software was applied to quantify the number of nodes, branches, and tubes.

### EC Vitality and Functionality in SCMO

Osteoblasts were cultured on the sample surface, and SCMO was harvested after culturing for 1 day. The SCMO was centrifuged and used to culture ECs. Then, VEGF secretion and the capillary tube formation of ECs were measured.

### The Culture of Macrophages

In this study, mouse RAW 264.7 cells (TIB-71, ATCC, Manassas, VA, United States) were used as the macrophage model. After culturing in standard medium overnight, lipopolysaccharide (1 μg/ml) was added to the medium to activate the macrophages. After activation for 2 h, the culture medium was renewed by serum-free medium, and the cells were cultured for another 6 h, followed by collection of SCMM that was centrifuged and stored at −80°C.

### Immune-Related and Osteogenic Gene Expression of Macrophage Culture on the Sample Surface

The total RNA was extracted from the macrophages using TRIzol (Life Technologies). Next, 1,200 ng of total RNA was reversely transcribed into cDNA *via* a SensiFAST^TM^ cDNA Synthesis Kit (Bioline, Australia). The expression levels of osteogenic-related genes [bone morphogenetic protein 2 (BMP-2), transforming growth factor-β (TGFβ), and VEGF], osteoclastic-related genes [tartrate-resistant acid phosphatase (TRAP) and cathepsin K (CTSK)], inflammatory cytokine genes [interleukin-1β (IL-1β), IL-6, IL-18, and tumor necrosis factor alpha (TNF-α)], macrophage surface marker genes [inducible nitric oxide synthase (iNOS), CD86, IL-10, CD163, and CD206], and autophagy-related genes [light chain 3A (LC3A), LC3B, P62, autophagy-related 5 (ATG5), and ATG7] were analyzed by Quant Studio^TM^ real-time quantitative polymerase chain reaction (RT-qPCR) (Applied Biosystems, Carlsbad, CA, United States) with SYBR Green as fluorescence indicator (Life Technologies).

### Evaluation of Activity of OBs and ECs Stimulated in the SCMM

The OBs were cultured in SCMM for 72 h. The total RNA was extracted and reverse-transcribed into cDNA as previously described. The expression levels of osteogenic-related genes [type 1 collagen (COL1), osteocalcin (OCN), osteoprotegerin (OPG), runt-related gene 2 (Runx2), osterix (OSX), ALP, BMP-2, TGF-β, and VEGFA] were analyzed by RT-qPCR. Meanwhile, the expression levels of angiogenetic-related genes [endothelial NOS (eNOS), von willebrand factor (vWF), platelet/endothelial cell adhesion molecule 1 (PECAM), VEGFA, angiopoietin-1 (ANG-1), fibroblast growth factors (FGF), and BMP-2] were also detected *via* RT-qPCR. The primer sequences for RT-qPCR are listed in Supplementary Table 1.

### Animal Model

Male New Zealand white rabbits (2.0–2.5 kg) were purchase from Shanghai SLAC Laboratory Animal Co., Ltd., China. All surgical procedures were approved by the Hangzhou Hibio Animal Care and Use Committee. General anesthesia was carried out *via* intramuscular injection, and surgical operations were performed under aseptic conditions. Operations were conducted on the distal surfaces of the bilateral femoral condyles, and composite materials (Ti–M, Ti–M–H1, Ti–M–H4, and Ti–M–H8) were prepared and inserted into the bone cavities with physiological saline. At 5 days after the operations, ampicillin sodium was administered, and after 8 weeks, all animals were sacrificed *via* air injection intravenously. The femurs and tibias of the four treatment groups were isolated, fixed in 4% PFA immediately, and dehydrated in gradient ethanol.

### μ-Computed Tomography Scanning

The 3D structure of dissected specimens was analyzed *via* μ-computed tomography (μ-CT) (Scanco Medical, Nokomis, FL, United States) with the parameters as follows: 70 kVp, 114 μA, and scanning thickness of 10 μm. The region of interest was selected, of which cortical bone was analyzed within 0.5 mm from the implant axis to the surface. A 3D model of the images was reconstructed and analyzed by the SCANCO software.

### Histological Analysis

The resin-embedded non-decalcified samples were sectioned using an EXAKT saw (EXAKT Apparatebau, Norderstedt, Germany), which were ground to a thickness of 50 μm *via* EXAKT-polish machine and stained using toluidine blue. Two sections of each group were selected to evaluate the histology and histomorphometry. The images were captured using Zeiss microscope (Zeiss). Bone–implant contact (BIC) was quantified from images photographed under × 5 magnification through Image J software. BIC is defined as the ratio of the total length of the implant that was directly in contact with the new bone tissue to the total length of the implant that was adjacent to the natural bone.

### Statistical Analysis

Statistical analysis was performed using SPSS 21.0 (IBM Ltd., Armonk, NY, United States). Quantitative data were presented as mean ± standard deviation. Data among multiple groups were processed by one-way analysis of variance and Tukey’s *post hoc* test. *p* < 0.05 indicated a statistically significant difference.

## Results

### Surface Morphology and Component Characterization of Ti–M–H

**FIGURE 1:**
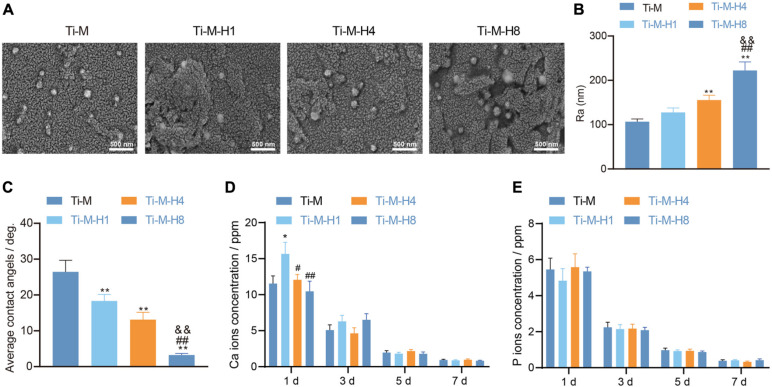
Surface morphology and component characterization of Ti–M–H coatings. **(A)** Coating morphology *via* scanning electron microscopy. **(B)** Roughness analysis on the coating surface. **(C)** Sample surface wettability test. **(D)** Amount of Ca ion release after 1 day of immersion in phosphate-buffered saline. **(E)** The amount of P ion released after 1 day of immersion in PBS. *P < 0.05 compared with Ti–M. ^#^*P* < 0.05 compared with Ti–M–H1. ^&^*P* < 0.05 compared with Ti–M–H4. **P < 0.01 compared with Ti–M. ^##^*P* < 0.01 compared with Ti–M–H1. ^&&^*P* < 0.01 compared with Ti–M–H4. The experiments were repeated three times.

To study the effect of Ti–M–H on osseointegration, the morphology of the Ti–M–H coating surface by MAO and SHT treatment is shown in Figure 1A. The data showed that MAO could produce a uniformly distributed volcanic porous structure on the Ti surface with an average diameter of 1.1 μm. Next, it was demonstrated that SHT treatment formed nano-structures on the surface of Ti. As the SHT time increased, the nano-particle size and roughness became larger with time extension; among them, the roughness of Ti–M–H8 was 2,050 nm, which was notably higher than that in other groups (Figure 1B). EDX mapping uncovered that the cross-sectional component of the composite coating consisted of Ca, P, Ti, and O that were homogeneously distributed (Supplementary Figure 1), and the coating surface becomes more hydrophilic (Figure 1C). Compared with Ti–M, the number of Ca ions released from the Ti–M–H1 surface was notably increased. The release of Ca ions on the surface of all samples was decreased with no significant difference in a time-dependent manner (Figure 1D). This was due to the fact that the nano-HA on the surface of Ti–M–H1 was much smaller than that of Ti–M–H4 and Ti–M–H8, which released Ca ions from the surface of Ti–M–H1. On the contrary, the P ions released among different groups showed no significant difference. The P ions released from all samples declined in a time-dependent manner (Figure 1E). The XRD pattern showed that Ti–M was composed of rare rutile and a large amount of anatase. Since the hydrothermal time was extended from 1 to 8 h, the characteristic HA peak became clearer, and the peak width narrowed (Supplementary Figure 2). This indicates that the HA crystallinity on the surface of the sample increased after hydrothermal treatment. Altogether the data illustrate that MAO and SHT produced microporous Ti samples deposited by nanoparticles.

### Ti–M–H1 Coating Promotes the Osteogenesis of MC3T3-E1 Cells

**FIGURE 2:**
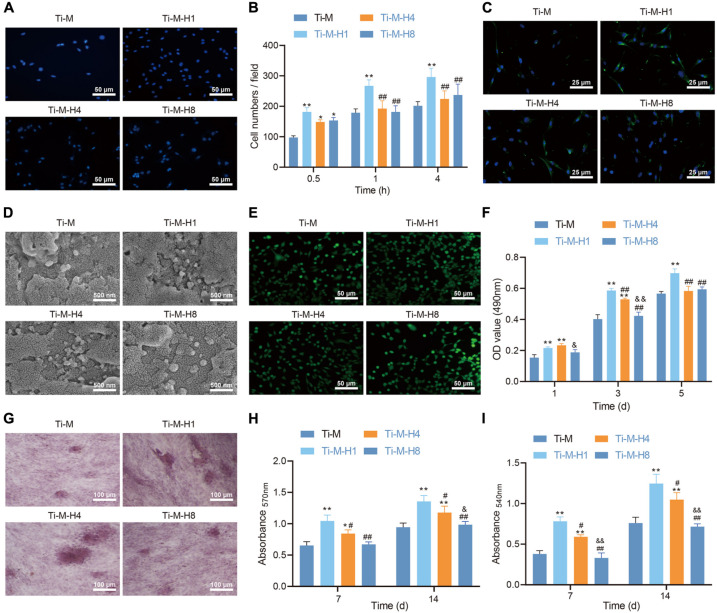
Ti–M–H coating promotes osteogenesis *in vitro*. **(A)** Representative images of osteoblasts that adhered to the coating surface for 0.5 h by 4,6-diamidino-2-phenylindole. **(B)** Quantitative results of MC3T3-E1 cells that adhered to the coating surface for 0.5, 1, and 4 h. **(C)** Fluorescent images of cell skeleton of MC3T3-E1 cells after 24 h of incubation on the sample surface. **(D)** Scanning electron microscopy images of MC3T3-E1 cells cultured on the coating surface for 1 day. **(E)** Live/dead images of MC3T3-E1 cells cultured on the sample surface for 1, 3, and 5 days. **(F)** MC3T3-E1 cell proliferation on the coating surface as determined by the 3-(4,5-dimethylthiazol-2-yl)-2,5-diphenyltetrazolium bromide method. **(G)** ALP staining images of MC3T3-E1 cells cultured on the coating surface for 7 days. **(H)** Quantification of collagen secretion of MC3T3-E1 cells cultured on the coating surface for 7 and 14 days. **(I)** Quantification of extracellular matrix mineralization of MC3T3-E1 cell cultured on the coating surface for 7 and 14 days. *P < 0.05 compared with Ti–M. ^#^*P* < 0.05 compared with Ti–M–H1. ^&^*P* < 0.05 compared with Ti–M–H4. **P < 0.01 compared with Ti–M. ^##^*P* < 0.01 compared with Ti–M–H1. ^&&^*P* < 0.01 compared with Ti–M–H4. The experiments were repeated three times.

To study the effect of Ti–M–H1 coating on osteogenesis, the function of the osteoblast cell line MC3T3-E1 on the surface of different coatings was detected. DAPI staining was performed to indicate the number of cells attached to the samples after incubation for 0.5, 1, and 4 h. The qualitative evaluation at 0.5 h demonstrated that the amount of cells adhesive on Ti–M–H was considerably increased than that on Ti–M (Figure 2A). Under longer incubation (1 and 4 h), only Ti–M–H1 maintained a relatively higher number of adhesive cells, while Ti–M, Ti–M–H4, and Ti–M–H8 displayed no significant differences (Figure 2B). Besides this, more F-actin syntheses were found in cells on Ti–M–H1 after 4 h of incubation in comparison to the other groups, which became more evident after 24 h of incubation (Figure 2C). In the meantime, the morphology analysis *via* SEM revealed that the MC3T3-E1 cells presented smooth pseudopodia and extension on Ti–M–H1 surface, and cell spreading on Ti–M–H4 and Ti–M–H8 surfaces was restricted, indicating that the deposition of large amounts of nano-HA on Ti–M–H4 and Ti–M–H8 may inhibit cell proliferation (Figure 2D). Next, live/dead staining analysis revealed no cytotoxicity of the four samples (Figure 2E). Ti–M–H1 displayed significant cell adhesion, spreading, and proliferation, which was also validated by the MTT assay (Figure 2F). ALP qualitative analysis demonstrated that Ti–M–H1 and Ti–M–H4 induced a higher ALP expression than that in other groups (Figure 2G). Similarly, compared with Ti–M, Ti–M–H1, and Ti–M–H4 remarkably enhanced collagen secretion and ECM mineralization after induction of osteogenesis for 7 and 14 days (Figures 2H,I). It can be concluded that, in comparison to Ti–M with the single microstructure, Ti–M–H1 with nanoparticle structure could promote cell adhesion and spreading of OBs and enhance differentiation.

### Ti–M–H Coating Promotes Angiogenesis *in vitro*

**FIGURE 3:**
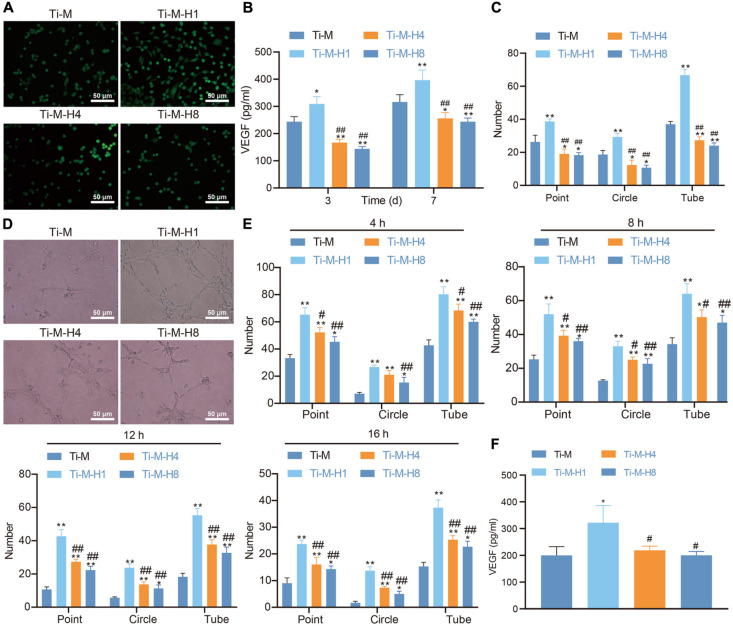
Ti–M–H coating promotes angiogenesis *in vitro*. **(A)** Live/dead images of endothelial cells (ECs) cultured on the sample surface for 1, 3, and 5 days. **(B)** The secretion of VEGF by ECs. **(C)** Quantitative: the number of nodes, branches, and tube formation formed in capillary-like networks after ECs were incubated in sample extract for 7 h. **(D)** Images of capillary-like networks by ECs incubated in SCMO for 4, 8, 12, and 16 h. **(E)** Quantitative: the number of nodes, branches, and tube formation after incubation for 4, 8, 12, and 16 h. **(F)** VEGF secretion after the ECs were incubated in SCMO for 8 days. *P < 0.05 compared with Ti–M. #*P* < 0.05 compared with Ti–M–H1. ^&^*P* < 0.05 compared with Ti–M–H4. **P < 0.01 compared with Ti–M. ^##^*P* < 0.01 compared with Ti–M–H1. ^&&^*P* < 0.01 compared with Ti–M–H4. The experiments were repeated three times.

Angiogenesis is another key factor of osseointegration. In this study, ECs were cultured on the surface of Ti–M–H to investigate its effect on angiogenesis. Live/dead staining was conducted to assess cell viability, which uncovered that all four Ti surfaces supported the adhesion and proliferation of ECs; Ti–M–H1 was especially the most effective for the adhesion and proliferation of ECs (Figure 3A). The VEGF level was determined 3 and 7 days after the implantation of ECs on the coating surface. At both time points, Ti–M–H1 induced a notably higher amount of VEGF secretion than that in Ti–M control; however, Ti–M–H4 and Ti–M–H8 remarkably decreased the secretion of VEGF (Figure 3B). Next, the qualitative and quantitative analyses uncovered that ECs grown in Ti–M–H1 medium produced a capillary-like network, and the number of nodes, branches, and tube formation was remarkably higher in comparison to those in other treatments, while Ti–M–H4 and Ti–M–H8 cannot induce the network formation by ECs and thus cannot promote angiogenesis (Figure 3C). It was reported that OBs can express angiogenic factors like VEGF and erythropoietin. Thus, the effect of SCMO on the angiogenesis of ECs was further investigated. The results demonstrated that the SCMO of Ti–M–H1 remarkably increased the capillary-like tube formation by quantifying the number of nodes, branches, and tube formation, which were similar to previous data (Figures 3D,E). Meanwhile, Ti–M–H1-derived SCMO considerably promoted the secretion of VEGF by ECs (Figure 3F). These results indicated that the Ti–M–H1 surface could promote angiogenesis.

### Ti–M–H1 Coating Promotes Osteogenesis/Angiogenesis by Regulating Immune Responses

**FIGURE 4:**
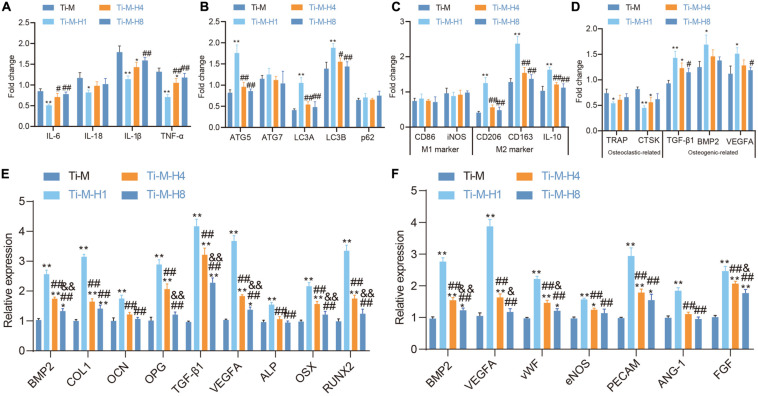
Ti–M–H coating on macrophage immunomodulation, osteogenesis, and angiogenesis. **(A)** RT-qPCR to detect the effect of coating surface on the expression of macrophage inflammatory factors. **(B)** RT-qPCR to detect the effect of coating surface on the expression of macrophage autophagy factors. **(C)** RT-qPCR to detect the effect of coating surface on the expression of M1 and M2 factors. **(D)** Effect of sample surface on macrophage osteoclast and osteogenic factors. **(E)** RT-qPCR to detect the expression of osteogenic-related genes in SCMM. **(F)** RT-qPCR to detect the expression of angiogenic-related genes in SCMM. *P < 0.05 compared with Ti–M. ^#^*P* < 0.05 compared with Ti–M–H1. ^&^*P* < 0.05 compared with Ti–M–H4. **P < 0.01 compared with Ti–M. ^##^*P* < 0.01 compared with Ti–M–H1. ^&&^*P* < 0.01 compared with Ti–M–H4. The experiments were repeated three times.

Recent studies illustrated that the osseointegration process of implants in the body is not only related to osteoblasts (Frosch et al., 2003), but the response of the immune system also has an important impact on the osseointegration behavior of materials (Trindade et al., 2018). Macrophages are the main effector cells of bone immune response, and their functional expression will affect the subsequent behavior of osteoblast lines. Macrophages were seeded on the Ti–M–H coating surface to study its role in immunomodulation; the gene expression patterns of macrophages were also studied, which demonstrated that Ti–M–H1 could sharply downregulate the expression of the pro-inflammatory cytokines IL1β, IL-6, IL-18, and TNF-α to inhibit inflammation (Figure 4A), promote the expression of autophagy markers LC3A, LC3B, and ATG5 on the sample surface (Figure 4B), and upregulate the expression of M2-type macrophage-related cytokines on the Ti–M–H1 surface notably (Figure 4C). Our data also revealed that Ti–M–H1 and Ti–M–H4 remarkably upregulated the expression levels of osteogenic markers (TGF-β1, BMP2, and VEGFA), while they downregulated the expression levels of osteoclastic markers (TRAP and CTSK) (Figure 4D). To investigate the effects of osteoimmunomodulation on osteogenesis and angiogenesis, the SCMM was collected and applied to culture OBs and ECs. First, OBs were cultured in the SCMM in order to analyze gene expression, which showed that Ti–M–H1 treatment dramatically upregulated the expressions of osteogenic-related genes, including BMP-2, COL1, OCN, OPG, TGF-β1, VEGFA, ALP, OSX, and RUNX2 (Figure 4E), and also notably upregulated the expression of angiogenic-related genes such as VEGFA, vWF, eNOS, PECAM, ANG-1, FGF, and BMP-2 (Figure 4F). The above-mentioned results supported that the Ti–M–H coating could regulate the immune response of macrophages and promote osteogenic differentiation and angiogenesis.

### Ti–M–H1 Coating Promotes Osseointegration of *in vivo* Implants

**FIGURE 5:**
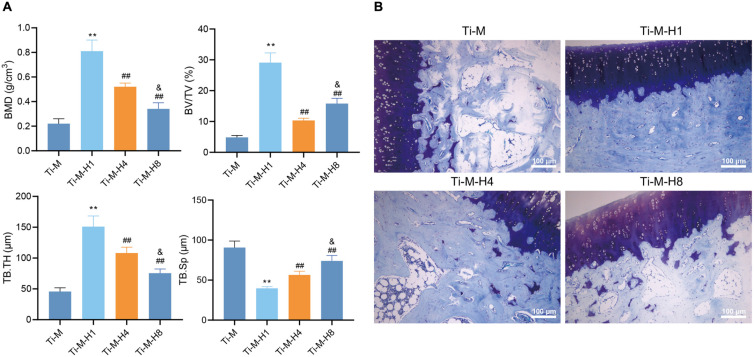
Osteogenesis of *in vivo* implants. **(A)** The micro-architectural parameters displayed by the three-dimensional reconstructed μCT image, including bone mineral density, bone volume fraction, and trabecular thickness, and trabecular separation. **(B)** Images of toluidine blue staining. *P < 0.05 compared with Ti–M. ^#^*P* < 0.05 compared with Ti–M–H1. ^&^*P* < 0.05 compared with Ti–M–H4. **P < 0.01 compared with Ti–M. ^##^*P* < 0.01 compared with Ti–M–H1. ^&&^*P* < 0.01 compared with Ti–M–H4. The experiments were repeated three times.

In order to verify that Ti–M–H1 coating can promote osseointegration *in vivo*, the prepared composite coatings (Ti–M, Ti–M–H1, Ti–M–H4, and Ti–M–H8) were inserted into the rabbit bone cavity. 3D reconstructions by μ-CT illustrated that Ti–M–H4 and Ti–M–H8 markedly increased the bone mineral density, bone volume fraction, and trabecular thickness but reduced trabecular separation (Figure 5A). The bone-to-implant interface was displayed by toluidine blue staining, which revealed that Ti–M–H1 remarkably enhanced the *de novo* bone formation (Figure 5B). Taken together, our data suggest that Ti–M–H1 can promote osseointegration.

## Discussion

In this paper, we demonstrate that the HA particle coated on the Ti surface *via* MAO and SHT favors the adhesion, proliferation, and differentiation of the OBs and ECs, which benefits osteogenesis, angiogenesis, and osseointegration. This nano-structure can also regulate the immune environment *in vivo* to enhance the communication between osteogenesis and angiogenesis and ultimately increase the process of osseointegration.

Micro-arc oxidation is an electrochemical process to generate microporous oxide coating on the surface of metals (Li et al., 2004, 2015). In the presence of HA, the Ti surface was coated with nano-HA. In the thermodynamic environment, SHT, Ca, and P are nucleated on the surface of the coatings, react with hydroxyl, and form fine crystals (Zhou et al., 2013, 2015). FE-SEM, EDX, XRD, Bruker stylus profiler, and Digimizer were carried out to characterize the morphology of modified Ti, which confirmed the composition of the surface with Ca, P, O, and Ti. We name it as Ti–M or correspondingly Ti–M–H1, Ti–M–H4, and Ti–M–H8 based on the different SHTs. Our data also show the nano-particle structures on Ti–M–H1, which switch to nano-rod structures on Ti–M–H4 and Ti–M–H8 surfaces, indicating crystal growth. After the immersion of Ti–M in DMEM for 3 days, nano-HA is completely covered by apatite that may be due to the existence of nano-HA. Increasing SHT time makes the coating surface more hydrophilic along with increasing crystal volume, which suggests that MAO and SHT can produce microporous Ti with nano-particle deposition, in accordance with a previous study (Bai et al., 2018).

Next, the current study further evaluated whether Ti–M–H affected osteogenesis, angiogenesis, and osteoimmunomodulation. First, our results uncover that nano-particle-structured Ti–M–H1 induces the adhesion, spreading, proliferation, and differentiation of OBs. However, nano-rod-structured Ti–M–H4 and H8 showed less effect on osteogenesis compared with Ti–M–H1. This is due to the fact that Ti–M–H1 provides a favorable environment, like pore size and HA crystallinity, that is close to the native bone physiochemical properties for OBs’ growth and differentiation (Wang et al., 2014; Zhou et al., 2014; Song et al., 2019). Angiogenesis is another indicator to assess successful osseointegration (Raines et al., 2019). OBs have been reported to produce VEGF-A, which is a key player in angiogenesis during bone formation and healing (Raines et al., 2019). Thus, we culture ECs either on the specimen surface or in SCMO to evaluate the capability of neovascularization. The data reveals that, in both cases, Ti–M–H1 induces higher EC adhesion and proliferation, upregulates VEGF secretion, and remarkably increases the formation of capillary-like network and the number of nodes, branches, and tube formation in comparison to Ti–M–H4 and H8.

VEGF is known as the key regulator of angiogenesis, which causes the production of nitric oxide (NO) (Melincovici et al., 2018). Meanwhile, NO diffusion promotes the adhesion, proliferation, and angiogenesis of ECs (Papapetropoulos et al., 1997). Therefore, our results indicated that Ti–M–H1 induces angiogenesis by upregulating VEGF and that VEGF secretion by OBs also contributed to neovascularization. The transplantation of Ti–M will reside in an immune microenvironment shared with native bones, in which macrophages are important mediators in the innate immune system and are involved in osteoimmunomodulation (Chen et al., 2017). Macrophages can be classified as M1 or M2 macrophage depending on their function and cytokine secretion (Tardito et al., 2019). M1 macrophages are pro-inflammatory cells promoting inflammation, whereas M2 macrophages suppress the production of pro-inflammatory cytokines and promote constructive tissue repair (Jablonski et al., 2015). Our data shows that Ti–M–H1 could promote the polarization of macrophages toward M2 and that Ti–M–H1 and Ti–M–H4 increase the expression levels of osteogenic markers (TGF-β1, BMP2, and VEGFA) but decrease the expression levels of osteoclastic markers (TRAP and CTSK), which together decrease inflammation while promoting osteogenesis.

To explore the interactions among osteoimmunomodulation, osteogenesis, and angiogenesis, we also culture OBs and ECs in the SCMM. Our gene expression results demonstrate that Ti–M–H1 is positively correlated with osteogenesis as seen by the upregulation of osteogenic-related genes including BMP-2, COL1, OCN, OPG, TGF-β1, VEGFA, ALP, OSX, and RUNX2. Our data also uncovers the upregulation of angiogenic-related genes such as VEGFA, vWF, eNOS, PECAM, ANG-1, FGF, and BMP-2, which indicates the role in angiogenesis *via* activating the VEGF signaling pathway. Finally, we validate the osseointegration of Ti–M–H1 *in vivo*. It is demonstrated that Ti–M–H1 promotes *de novo* bone formation, increases bone-to-implant interface, and provides attractive substrate for the dendrites, which confirms the osseointegration property of Ti–M–H1 *in vivo*.

When compared with the previously reported nanoTiO_2_/Ti-2448 (Liu et al., 2015), Ti–M–H1 has obvious advantages (Supplementary Table 2). The surface of Ti–M–H1 contains volcanic pores with an average diameter of 1.1 μm, while the surface of nanoTiO_2_/Ti-2448 mainly has 2-μm-wide grooves. Ti–M–H1 is mainly composed of Ti, O, Ca, and P, while nanoTiO_2_/Ti-2448 is mainly composed of Ti, O, Ca, and Zr. Ti–M–H1 can promote the adhesion and proliferation of MC3T3-E1 cells within 0.5–4 h and does not produce cytotoxicity after 5 days of culture. Meanwhile, nanoTiO_2_/Ti-2448 promotes the proliferation of MC3T3-E1 within 72 h without cytotoxicity. Ti–M–H1 can induce a high expression of ALP and significantly enhance collagen secretion and matrix mineralization after 7 and 14 days of osteogenic induction. NanoTiO_2_/Ti-2448 and MC3T3-E1 cultured for 3 or 5 days also induce a high ALP expression. Ti–M–H1 can promote endothelial cell angiogenesis and VEGF secretion. Ti–M–H1 can regulate the immune response of macrophages, promote the secretion of osteoblast factors, and inhibit the expression of osteoclast factors. Ti–M–H1 can significantly enhance the formation of new bone in the bone cavity after 8 weeks of surgery. The dense connective tissue around nanoTiO_2_/Ti-2448 is tighter at 12 weeks after surgery, and no signs of infection are observed around the implant.

## Conclusion

**FIGURE 6:**
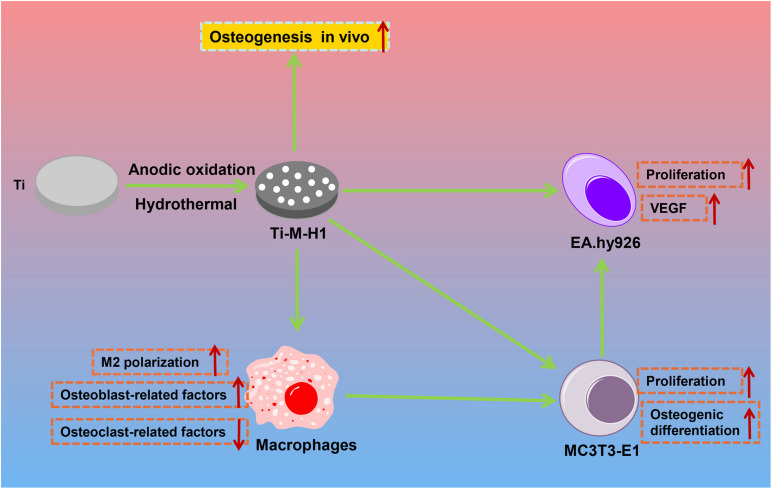
The mechanism graph of the nano-HA forms nanoparticles or nano-rods onto the microporous Ti surfaces. The nano-HA-coated Ti–M–H1 not only induces the osteogenesis and angiogenesis *in vitro* but also enhances the polarization of macrophage toward M2 to regulate the osteoimmune microenvironment.

In conclusion, this study successfully produces a composite bioactive coating with a micro–nano structure by MAO and SHT. By controlling the SHT processing time, the nano-HA forms nanoparticles or nano-rods onto the microporous Ti surfaces. The nano-HA-coated Ti–M–H1 not only induces osteogenesis and angiogenesis *in vitro* but also enhances the polarization of macrophages toward M2 to regulate the osteoimmune microenvironment (Figure 6), which will promote the communication between osteogenesis and angiogenesis, and ultimately accelerates the process of osseointegration *in vivo*.

## Data Availability Statement

The original contributions presented in the study are included in the article/Supplementary Material, further inquiries can be directed to the corresponding author/s.

## Ethics Statement

The animal study was reviewed and approved by the Hangzhou Hibio Animal Care and Use Committee.

## Author Contributions

XW, XJ, and JL designed the study. LM, XL, ZM, and FW collated the data, carried out the data analyses, and produced the initial draft of the manuscript. MJ, YX, and JZ contributed to drafting of the manuscript. All authors have read and approved the final submitted manuscript.

## Conflict of Interest

The authors declare that the research was conducted in the absence of any commercial or financial relationships that could be construed as a potential conflict of interest.

## Publisher’s Note

All claims expressed in this article are solely those of the authors and do not necessarily represent those of their affiliated organizations, or those of the publisher, the editors and the reviewers. Any product that may be evaluated in this article, or claim that may be made by its manufacturer, is not guaranteed or endorsed by the publisher.

## Supplementary Material

Supplementary Figure 1 Energy-dispersive X-ray total spectrum of the samples.

Supplementary Figure 2 X-ray diffraction pattern of the samples.

Supplementary Table 1 Primer sequences for RT-qPCR.

Supplementary Table 2 Comparison of osteogenesis function between Ti–M–H1 and NanoTiO_2_/Ti-2448.

## References

[B1] Bai L. Liu Y. Du Z. Weng Z. Yao W. Zhang X. (2018). Differential effect of hydroxyapatite nano-particle versus nano-rod decorated titanium micro-surface on osseointegration. *Acta Biomater.* 76 344–358. 10.1016/j.actbio.2018.06.023 29908975

[B2] Brydone A. S. Meek D. Maclaine S. (2010). Bone grafting, orthopaedic biomaterials, and the clinical need for bone engineering. *Proc. Inst. Mech. Eng. H* 224 1329–1343. 10.1243/09544119JEIM770 21287823

[B3] Chen Z. Bachhuka A. Wei F. Wang X. Liu G. Vasilev K. (2017). Nanotopography-based strategy for the precise manipulation of osteoimmunomodulation in bone regeneration. *Nanoscale* 9 18129–18152. 10.1039/c7nr05913b 29143002

[B4] Fillingham Y. Jacobs J. (2016). Bone grafts and their substitutes. *Bone Joint J.* 98-B 6–9. 10.1302/0301-620X.98B.36350 26733632

[B5] Frosch K. H. Sondergeld I. Dresing K. Rudy T. Lohmann C. H. Rabba J. (2003). Autologous osteoblasts enhance osseointegration of porous titanium implants. *J. Orthop. Res.* 21 213–223. 10.1016/S0736-0266(02)00143-2 12568951

[B6] Gulick D. T. Yoder H. N. (2002). Anterior cruciate ligament reconstruction: clinical outcomes of patella tendon and hamstring tendon grafts. *J. Sports Sci. Med.* 1 63–71. 24701126 PMC3967431

[B7] Jablonski K. A. Amici S. A. Webb L. M. Ruiz-Rosado Jde D. Popovich P. G. Partida-Sanchez S. (2015). Novel Markers to Delineate Murine M1 and M2 Macrophages. *PLoS One* 10:e0145342. 10.1371/journal.pone.0145342 26699615 PMC4689374

[B8] Jiang N. Guo Z. Sun D. Ay B. Li Y. Yang Y. (2019). Exploring the mechanism behind improved osteointegration of phosphorylated titanium implants with hierarchically structured topography. *Colloids Surf. B Biointerfaces* 184:110520. 10.1016/j.colsurfb.2019.110520 31590052

[B9] Kao R. T. Conte G. Nishimine D. Dault S. (2005). Tissue engineering for periodontal regeneration. *J. Calif. Dent. Assoc.* 33 205–215. 15918402

[B10] Khademhosseini A. Langer R. (2016). A decade of progress in tissue engineering. *Nat. Protoc.* 11 1775–1781. 10.1038/nprot.2016.123 27583639

[B11] Lavender C. Sina Adil S. A. Singh V. Berdis G. (2020). Autograft Cartilage Transfer Augmented With Bone Marrow Concentrate and Allograft Cartilage Extracellular Matrix. *Arthrosc. Tech.* 9 e199–e203. 10.1016/j.eats.2019.09.022 32099772 PMC7029053

[B12] Li L. H. Kong Y. M. Kim H. W. Kim Y. W. Kim H. E. Heo S. J. (2004). Improved biological performance of Ti implants due to surface modification by micro-arc oxidation. *Biomaterials* 25 2867–2875. 10.1016/j.biomaterials.2003.09.048 14962565

[B13] Li Z. Yi J. Huang B. Wu X. Qiao W. Luo X. (2015). Ultraviolet irradiation enhanced bioactivity and biological response of mesenchymal stem cells on micro-arc oxidized titanium surfaces. *Dent. Mater. J.* 34 135–147. 10.4012/dmj.2014-125 25736258

[B14] Liu P. Hao Y. Zhao Y. Yuan Z. Ding Y. Cai K. (2017). Surface modification of titanium substrates for enhanced osteogenetic and antibacterial properties. *Colloids Surf. B Biointerfaces* 160 110–116. 10.1016/j.colsurfb.2017.08.044 28918187

[B15] Liu X. H. Wu L. Ai H. J. Han Y. Hu Y. (2015). Cytocompatibility and early osseointegration of nanoTiO2-modified Ti-24 Nb-4 Zr-7.9 Sn surfaces. *Mater. Sci. Eng. C Mater. Biol. Appl.* 48 256–262. 10.1016/j.msec.2014.12.011 25579921

[B16] Luo J. P. Huang Y. J. Xu J. Y. Sun J. F. Dargusch M. S. Hou C. H. (2020). Additively manufactured biomedical Ti-Nb-Ta-Zr lattices with tunable Young’s modulus: mechanical property, biocompatibility, and proteomics analysis. *Mater. Sci. Eng. C Mater. Biol. Appl.* 114:110903. 10.1016/j.msec.2020.110903 32994002

[B17] Malhotra A. Habibovic P. (2016). Calcium Phosphates and Angiogenesis: implications and Advances for Bone Regeneration. *Trends Biotechnol.* 34 983–992. 10.1016/j.tibtech.2016.07.005 27481474

[B18] Melincovici C. S. Bosca A. B. Susman S. Marginean M. Mihu C. Istrate M. (2018). Vascular endothelial growth factor (VEGF) - key factor in normal and pathological angiogenesis. *Rom. J. Morphol. Embryol.* 59 455–467. 30173249

[B19] Mendonca G. Mendonca D. B. Simoes L. G. Araujo A. L. Leite E. R. Duarte W. R. (2009). The effects of implant surface nanoscale features on osteoblast-specific gene expression. *Biomaterials* 30 4053–4062. 10.1016/j.biomaterials.2009.04.010 19464052

[B20] Oryan A. Alidadi S. Moshiri A. Maffulli N. (2014). Bone regenerative medicine: classic options, novel strategies, and future directions. *J. Orthop. Surg. Res* 9:18. 10.1186/1749-799X-9-18 24628910 PMC3995444

[B21] Oryan A. Kamali A. Moshiri A. Baghaban Eslaminejad M. (2017). Role of Mesenchymal Stem Cells in Bone Regenerative Medicine: what Is the Evidence? *Cells Tissues Organs* 204 59–83. 10.1159/000469704 28647733

[B22] Papapetropoulos A. Garcia-Cardena G. Madri J. A. Sessa W. C. (1997). Nitric oxide production contributes to the angiogenic properties of vascular endothelial growth factor in human endothelial cells. *J. Clin. Invest.* 100 3131–3139. 10.1172/JCI119868 9399960 PMC508526

[B23] Patlolla A. Arinzeh T. L. (2014). Evaluating apatite formation and osteogenic activity of electrospun composites for bone tissue engineering. *Biotechnol. Bioeng.* 111 1000–1017. 10.1002/bit.25146 24264603

[B24] Pierre C. Bertrand G. Rey C. Benhamou O. Combes C. (2019). Calcium phosphate coatings elaborated by the soaking process on titanium dental implants: surface preparation, processing and physical-chemical characterization. *Dent. Mater.* 35 e25–e35. 10.1016/j.dental.2018.10.005 30424917

[B25] Raines A. L. Berger M. B. Patel N. Hyzy S. L. Boyan B. D. Schwartz Z. (2019). VEGF-A regulates angiogenesis during osseointegration of Ti implants via paracrine/autocrine regulation of osteoblast response to hierarchical microstructure of the surface. *J. Biomed. Mater. Res. A* 107 423–433. 10.1002/jbm.a.36559 30461195 PMC6892345

[B26] Shbeh M. Wally Z. J. Elbadawi M. Mosalagae M. Al-Alak H. Reilly G. C. (2019). Incorporation of HA into porous titanium to form Ti-HA biocomposite foams. *J. Mech. Behav. Biomed. Mater.* 96 193–203. 10.1016/j.jmbbm.2019.04.043 31054514

[B27] Sobolev A. Wolicki I. Kossenko A. Zinigrad M. Borodianskiy K. (2018). Coating Formation on Ti-6Al-4V Alloy by Micro Arc Oxidation in Molten Salt. *Materials* 11:611. 10.3390/ma11091611 30181496 PMC6163964

[B28] Sohn H. S. Oh J. K. (2019). Review of bone graft and bone substitutes with an emphasis on fracture surgeries. *Biomater. Res.* 23:9. 10.1186/s40824-019-0157-y 30915231 PMC6417250

[B29] Song P. Hu C. Pei X. Sun J. Sun H. Wu L. (2019). Dual modulation of crystallinity and macro-/microstructures of 3D printed porous titanium implants to enhance stability and osseointegration. *J. Mater. Chem. B* 7 2865–2877. 10.1039/c9tb00093c 32255089

[B30] Tardito S. Martinelli G. Soldano S. Paolino S. Pacini G. Patane M. (2019). Macrophage M1/M2 polarization and rheumatoid arthritis: a systematic review. *Autoimmun. Rev.* 18:102397. 10.1016/j.autrev.2019.102397 31520798

[B31] Trindade R. Albrektsson T. Galli S. Prgomet Z. Tengvall P. Wennerberg A. (2018). Bone Immune Response to Materials, Part I: titanium, PEEK and Copper in Comparison to Sham at 10 Days in Rabbit Tibia. *J. Clin. Med.* 7:526. 10.3390/jcm7120526 30544551 PMC6307090

[B32] Wang P. Zhao L. Liu J. Weir M. D. Zhou X. Xu H. H. (2014). Bone tissue engineering via nanostructured calcium phosphate biomaterials and stem cells. *Bone Res.* 2:14017. 10.1038/boneres.2014.17 26273526 PMC4472121

[B33] Wang X. Li B. Zhang C. (2019). Preparation of BMP-2/chitosan/hydroxyapatite antibacterial bio-composite coatings on titanium surfaces for bone tissue engineering. *Biomed. Microdevices* 21:89. 10.1007/s10544-019-0437-2 31655887

[B34] Wobma H. Vunjak-Novakovic G. (2016). Tissue Engineering and Regenerative Medicine 2015: a Year in Review. *Tissue Eng. Part B Rev.* 22 101–113. 10.1089/ten.TEB.2015.0535 26714410 PMC4817587

[B35] Yu B. Y. Zheng J. Chang Y. Sin M. C. Chang C. H. Higuchi A. (2014). Surface zwitterionization of titanium for a general bio-inert control of plasma proteins, blood cells, tissue cells, and bacteria. *Langmuir* 30 7502–7512. 10.1021/la500917s 24913288

[B36] Zhou H. Z. Li Y. D. Liu L. Chen X. D. Wang W. Q. Ma G. W. (2017). Early osseointegration of implants with cortex-like TiO2 coatings formed by micro-arc oxidation: a histomorphometric study in rabbits. *J. Huazhong Univ. Sci. Technol. Med. Sci.* 37 122–130. 10.1007/s11596-017-1705-0 28224420

[B37] Zhou J. Li B. Lu S. Zhang L. Han Y. (2013). Regulation of osteoblast proliferation and differentiation by interrod spacing of Sr-HA nanorods on microporous titania coatings. *ACS Appl. Mater. Interfaces* 5 5358–5365. 10.1021/am401339n 23668394

[B38] Zhou R. Wei D. Cao J. Feng W. Cheng S. Du Q. (2015). The effect of NaOH concentration on the steam-hydrothermally treated bioactive microarc oxidation coatings containing Ca, P, Si and Na on pure Ti surface. *Mater. Sci. Eng. C Mater. Biol. Appl.* 49 669–680. 10.1016/j.msec.2015.01.062 25686996

[B39] Zhou R. Wei D. Cheng S. Feng W. Du Q. Yang H. (2014). Structure, MC3T3-E1 cell response, and osseointegration of macroporous titanium implants covered by a bioactive microarc oxidation coating with microporous structure. *ACS Appl. Mater. Interfaces* 6 4797–4811. 10.1021/am405680d 24579697

