# Supplementary figures and images for: Hydroxyapatite-Coated Titanium by Micro-Arc Oxidation and Steam–Hydrothermal Treatment Promotes Osseointegration
Source: Front Bioeng Biotechnol. 2021 Aug 19;9:625877. doi: 10.3389/fbioe.2021.625877 (PMC8417371; doi:10.3389/fbioe.2021.625877)

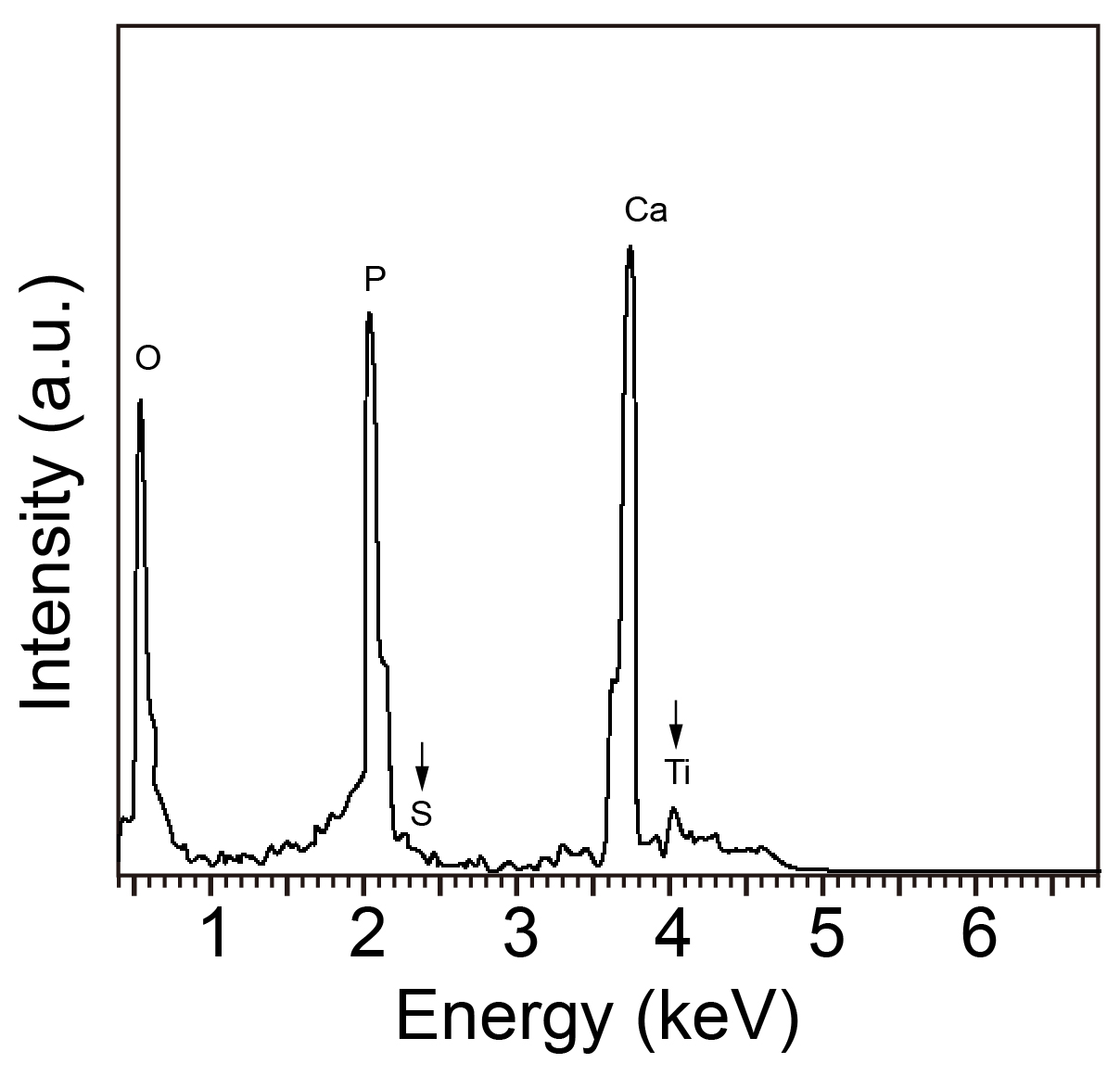

Supplement: Supplementary Figure 1 — Energy-dispersive X-ray total spectrum of the samples. [file Image_1.JPEG]

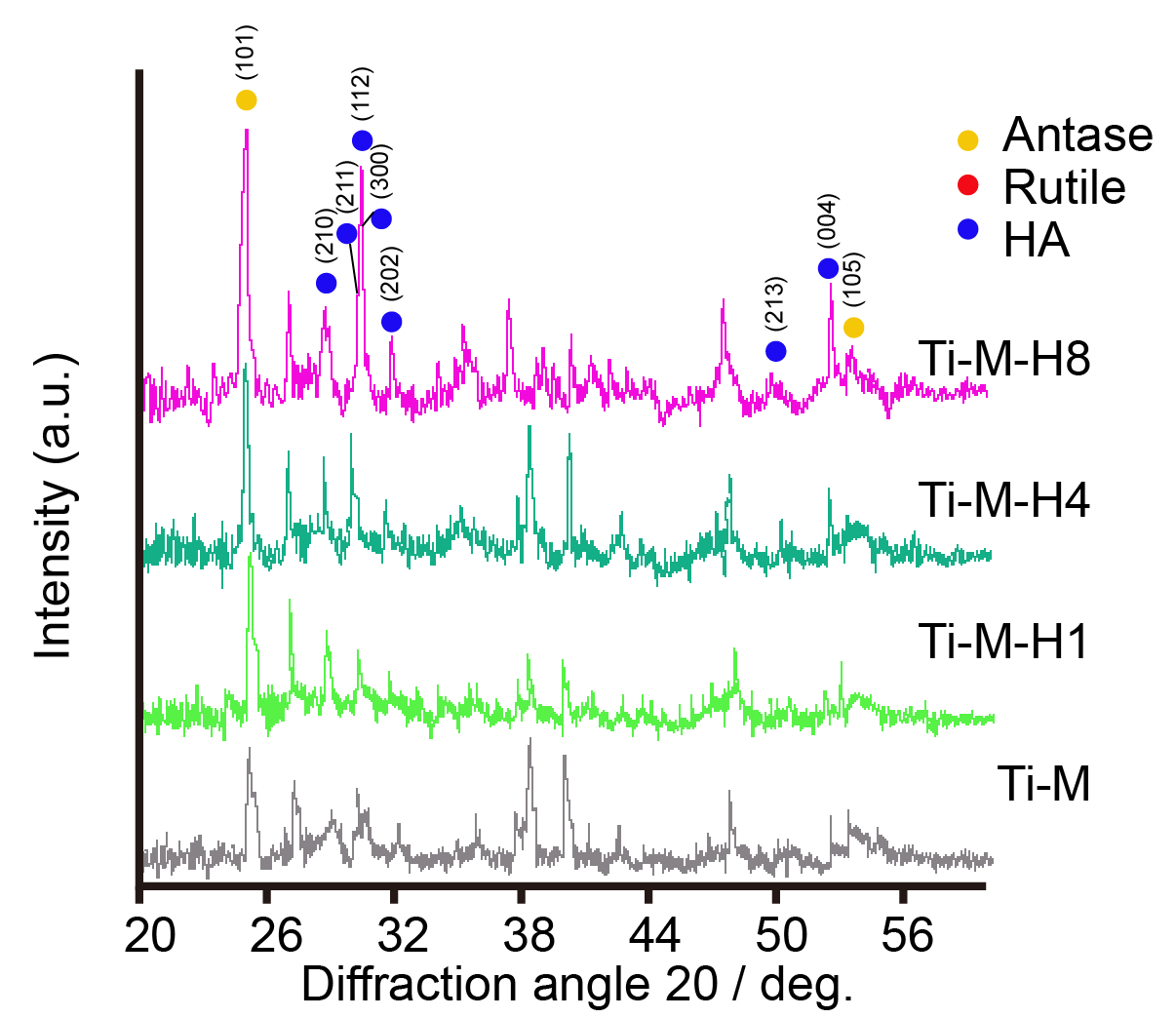

Supplement: Supplementary Figure 2 — X-ray diffraction pattern of the samples. [file Image_2.JPEG]
