# Supplementary material for: Hydroxyapatite-Coated Titanium by Micro-Arc Oxidation and Steam–Hydrothermal Treatment Promotes Osseointegration
Source: Front Bioeng Biotechnol. 2021 Aug 19;9:625877. doi: 10.3389/fbioe.2021.625877 (PMC8417371; doi:10.3389/fbioe.2021.625877)
Supplement: Supplementary Table 1 — Primer sequences for RT-qPCR. [file Table_1.docx]

**Supplementary table 1**. Primer sequences for RT-qPCR.

| **Genes** | **Primer sequences** |
| --- | --- |
| **Mouse species** | |
| IL-6 | Forward: 5’-ATAGTCCTTCCTACCCCAATTTCC-3’  Reverse: 5’-GATGAATTGGATGGTCTTGGTCC-3’ |
| IL-18 | Forward: 5’-TGGCCGACTTCACTGTACAAC-3’  Reverse: 5’-TGGGGTTCACTGGCACTTTG-3’ |
| IL-1β | Forward: 5’-TGGAGAGTGTGGATCCCAAG-3’  Reverse: 5’-GGTGCTGATGTACCAGTTGG-3’ |
| TNFα | Forward: 5’-CTGAACTTCGGGGTGATCGG-3’  Reverse: 5’-GGCTTGTCACTCGAATTTTGAGA-3’ |
| CD86 | Forward: 5’-CTGCTCATCATTGTATGTCAC-3’  Reverse: 5’-ACTGCCTTCACTCTGCATTTG-3’ |
| iNOS | Forward: 5’-CACCAAGCTGAACTTGAGCG-3’  Reverse: 5’-CGTGGCTTTGGGCTCCTC-3’ |
| CD206 | Forward: 5’-AGACGAAATCCCTGCTACTG-3’  Reverse: 5’-CACCCATTCGAAGGCATTC-3’ |
| CD163 | Forward 5’-CGTGTGCAGTGTCCAAAAGG-3’  Reverse 5’-CACAAACCAAGAGTGCCGTG-3’ |
| IL-10 | Forward 5’-GAGAAGCATGGCCCAGAAATC-3’  Reverse 5’-GAGAAATCGATGACAGCGCC-3’ |
| LC3A | Forward: 5’-ACAGCATGGTGAGCGTCTC-3’  Reverse: 5’-AGGTTTCTTGGGAGGCGTAG-3’ |
| LC3B | Forward: 5’-GATAATCAGACGGCGCTTGC-3’  Reverse: 5’-TCTCACTCTCGTACACTTCGG-3’ |
| ATG5 | Forward: 5’-GATGCGGTTGAGGCTCAC-3’  Reverse: 5’-CTGTCATTCTGCAGTCCCATC-3’ |
| ATG7 | Forward: 5’-AGCCTGTTCACCCAAAGTTC-3’  Reverse: 5’-CATGTCCCAGATCTCAGCAG-3’ |
| P62 | Forward: 5’-AGCTGCTCTTCGGAAGTCAG-3’  Reverse: 5’-CTCCATCTGTTCCTCTGGCTG-3’ |
| BMP2 | Forward: 5’-GCTCCACAAACGAGAAAAGC-3’  Reverse: 5’-AGCAAGGGGAAAAGGACACT-3’ |
| TGFβ1 | Forward: 5'- CAGTACAGCAAGGTCCTTGC-3'  Reverse: 5'- ACGTAGTAGACGATGGGCAG-3' |
| VEGFA | Forward: 5’-GTCCCATGAAGTGATCAAGTTC-3’  Reverse: 5’-TCTGCATGGTGATGTTGCTCTCTG-3’ |
| TRAP | Forward: 5’-CACTCCCACCCTGAGATTTGT-3’  Reverse: 5’-CATCGTCTGCACGGTTCTG-3’ |
| CTSK | Forward: 5’-CTGGAGGGCCAACTCAAGAA-3’  Reverse: 5’-TCCGTTCTGCTGCACGTATT-3’ |
| COL1 | Forward: 5’-CTCCGGCTCCTGCTCCTCTTA-3’  Reverse: 5’-ACCAGGAAGTCCAGGCTGTC-3’ |
| OCN | Forward: 5′-CCGGGAGCAGTGTGAGCTTA-3′  Reverse: 5′-AGGCGGTCTTCAAGCCATACT-3′ |
| OPG | Forward: 5’-CCTAAAGCGTTAACCCCGGA-3’  Reverse: 5’-AACAGGAAGTATGCCCTGCC-3’ |
| ALP | Forward: 5′-GGGCCTGCTCTGTTTCTTCA-3′  Reverse: 5′-CTGAGATTCGTCCCTCGCTG-3′ |
| OSX | Forward: 5′-CCCTTCTCAAGCACCAATGG-3′  Reverse: 5′-AGGGTGGGTAGTCATTTGCATAG-3′ |
| RUNX2 | Forward: 5′-AAATGCCTCCGCTGTTATGAA-3′  Reverse: 5′-GCTCCGGCCCACAAATCT-3′ |
| GAPDH | Forward: 5’-TGACCACAGTCCATGCCATC-3’  Reverse: 5’-GACGGACACATTGGGGGTAG-3’ |
| **Human species** | |
| VEGFA | Forward: 5’-TTAAACGAACGTACTTGCAGATG-3’  Reverse: 5’-GAGAGATCTGGTTCCCGAAA-3’ |
| vWF | Forward: 5’-CCCCTGAAGCCCCTCCTCCTA-3’  Reverse: 5’-ACGAACGCCACATCCAGAACC-3’ |
| eNOS | Forward: 5’-TCTTCCTGGACATCACCTCC-3’  Reverse: 5’-CTTCCACTCCTCGTAGCGTC-3’ |
| PECAM | Forward: 5’-CAACGAGAAAATGTCAGA-3’  Reverse: 5’-GGAGCCTTCCGTTCTAGAGT-3’ |
| ANG-1 | Forward: 5’-GTGCTGGGTCTGGGTCTGAC-3’  Reverse: 5’-GGCCTTGATGCTGCGCTTG-3’ |
| FGF | Forward: 5’-CTGTACTGCAAAAACGGG-3’  Reverse: 5’-AAAGTATAGCTTTCTGCC-3’ |
| BMP2 | Forward: 5’-TGGCCCACTTGGAGGAGAAACA-3’  Reverse: 5’-CGCTGTTTGTGTTTGGCTTGACG-3’ |
| GAPDH | Forward: 5′-CGGAGTCAACGGATTTGGTCGTAT-3’  Reverse: 5′-AGCCTTCTCCATGGTGGTGAAGAC-3’ |
