# Supplementary material for: Hydroxyapatite-Coated Titanium by Micro-Arc Oxidation and Steam–Hydrothermal Treatment Promotes Osseointegration
Source: Front Bioeng Biotechnol. 2021 Aug 19;9:625877. doi: 10.3389/fbioe.2021.625877 (PMC8417371; doi:10.3389/fbioe.2021.625877)
Supplement: Supplementary Table 2 — Comparison of osteogenesis function between Ti–M–H1 and NanoTiO2/Ti-2448. [file Table_2.DOCX]

**Supplementary Table 2** Comparison of osteogenesis function between Ti-M-H1 and NanoTiO2/Ti-2448

|  | Ti-M-H1 | NanoTiO_2_/Ti-2448 |
| --- | --- | --- |
| Sample surface | 1 μm volcanic pore size | 2 μm concave |
| Elements | Ti, O, Ca and P | Ti, O, Ca and Zr |
| Effects on MC3T3-E1 cells | Promoting the adhesion and proliferation of MC3T3-E1 cells within 0.5-4 h;  no cytotoxicity observed after 5 days of culture | Promoting the proliferation of MC3T3-E1 cells within 72 h without cytotoxicity |
| ALP activity, collagen deposition and matrix mineralization | Inducing ALP activity, collagen deposition and matrix mineralization after 7 and 14 days of osteogenic induction | Inducing ALP activity following culture for 3-5 days |
| EC angiogenesis and release of osteogenic factor | Promoting EC angiogenesis and VEGF secretion; Regulating immune response of macrophages;  Promoting osteogenic differentiation and angiogenesis; Inhibiting release of osteoclast factors | None |
| Osteogenesis *in vivo* | Enhancing the *de novo* bone formation in rabbit bone cavity 8 weeks after surgery | Inducing denser surrounding connective tissues without infection were observed around the implant 12 weeks after surgery |
